# 5'-Noraristeromycin Repurposing: Well-known S-Adenosyl-L-homocysteine Hydrolase Inhibitor As a Potential Drug Against Leukemia

**DOI:** 10.32607/actanaturae.27443

**Published:** 2024

**Authors:** O. N. Novikova, E. S. Matyugina, A. V. Gorshenin, Yu. I. Velikorodnaya, M. D. Krengauz, V. O. Vedernikova, P. V. Spirin, V. S. Prassolov, S. N. Kochetkov, A. L. Khandazhinskaya

**Affiliations:** Research Institute of Hygiene, Toxicology and Occupational Pathology, Federal Medical and Biological Agency, Volgograd, 400048 Russian Federation; Engelhardt Institute of Molecular Biology, Russian Academy of Sciences, Moscow, 119991 Russian Federation; Moscow Institute of Physics and Technology (National Research University), Dolgoprudny, 141701 Russian Federation; Center for High-Precision Editing and Genetic Technologies for Biomedicine, Institute of Molecular Biology. V.A. Engelhardt, Russian Academy of Sciences, Moscow, 119991 Russian Federation

**Keywords:** 5'-noraristeromycin, leukemia, cytotoxicity, acute toxicity, toxicometric parameters

## Abstract

5′-Noraristeromycin as a racemic mixture of enantiomers was found to exhibit a pronounced cytotoxic effect on leukemia cells; IC_50_ for the Jurkat, K562, and THP-1 cell lines was 7.3, 1.3, and 3.7 μM, respectively. The general toxicity of 5'-noraristeromycin was studied in experiments on white mice upon single-dose intragastric administration; toxicometric parameters were determined, and the clinical and pathomorphological presentation of acute intoxication was studied. LD_50_ of the substance was shown to be 63.2 (52.7÷75.8) mg/kg; LD16, 44.7 mg/kg, and LD84, 89.4 mg/kg. Administration of the substance at a dose within the studied dose range is accompanied by systemic damage to the internal organs and tissues of the experimental animals.

## INTRODUCTION

Drug repurposing for cancer therapy implies the search for compounds exhibiting an antitumor activity among substances used in the therapy of other diseases. This approach significantly reduces the cost of designing new drugs, since the substances have been well-studied and their manufacturing technologies have already been developed.

S-Adenosylhomocysteine hydrolase (SAH hydrolase) catalyzes the hydrolysis of SAH to adenosine and *L*-homocysteine, resulting in the accumulation of SAH, a natural inhibitor of S-adenosylmethioninedependent methyltransferases in the cell. SAH hydrolase inhibitors exhibit a strong antiviral activity, which is described using the terms of inhibiting maturation of viral mRNA (5'-capping) [1]. The SAH hydrolase gene is often amplified in malignant human neoplasms, including cervical and colon cancer [2, 3], indicating that SAH hydrolase can be used as a therapeutic target. Accumulation of SAH in eukaryotic cells treated with SAH hydrolase inhibitors, as well as altering the SAM/SAH ratio, was found to have numerous implications. First, it disrupts DNA methylation, which is a factor responsible for the epigen etic regulation of eukaryotic gene expression. DNA methylation disruption is revealed in cancer patients: while the overall genome is hypomethylated, the promoter regions of tumor suppressor genes are locally hypermethylated [4]. Second, it disturbs the function of PRC2 (Polycomb repressive complex 2), the conserved protein complex needed to maintain gene repression. The catalytic subunit of PRC2, the EZH2 protein, ensures the mono-, di-, and trimethylation of histone H3 (Lys27). A number of human tumors have been shown to be characterized by overexpression of PRC2 subunits and to carry mutations that enhance the catalytic activity of EZH2.

The antitumor activity of a number of SAH hydrolase inhibitors (e.g., neplanocin A, 3-deazaneplanocin, 3-deazaadenosine, aristeromycin, etc.) has been demonstrated on different tumor cell lines and even *in vivo *[5, 6, 7, 8, 9]. Aristeromycin was first isolated from a *Streptomyces citricolor *culture in 1967 [10]; synthesis of its derivative, 5'-norasteromycin, from 5-amino-4,6-dichloropyrimidine, was reported in 1992 [11]. In *in vitro *experiments, this compound exhibited a pronounced antiviral activity against cowpox, smallpox, and vesicular stomatitis viruses, parainfluenza virus type 3, reovirus type 1, human cytomegalovirus, as well as hepatitis B, measles, and influenza B viruses [12, 13, 14]. The antiviral properties of 5'-norasteromycin are based on the inhibition of S-adenosyl-*L*-homocysteine hydrolase activity [12, 14]. This compound was found to highly selectively suppress, along with S-adenosyl-*L*-homocysteine hydrolase, the activity of alpha subunit IκB kinase, the key kinase involved in the nuclear factor kappa B activation cascade [15]. The pharmacokinetic parameters of 5'-noraristeromycin administered orally at a dose of 10 mg/kg were determined in the same study; its prophylactic and therapeutic effects associated with the inhibition of tumor necrosis factor α at a dose of 1 mg/kg were revealed using a rheumatoid arthritis model. Based on computer simulation data, Singh et al. [16] hypothesized that this compound may be hepatotoxic. The earliest data on the cytostatic properties of 5'-noraristeromycin were published 30 years ago [12]; at concentrations of 0.39–0.50 μg/mL it inhibited proliferation in cultures of L1210/0 mouse leukemia cells as well as human lymphocytes Molt4 and CEM/0. However, comprehensive studies of the substance's cytotoxicity have not been conducted yet and we were unable to find any publications that assess the degree of acute toxicity of this substance to animals.

The objective of this study was to evaluate the antitumor activity of 5'-noraristeromycin in cell cultures, followed by an investigation of the characteristics of its toxicity in homeothermic animals and assessment of its main toxicometric parameters.

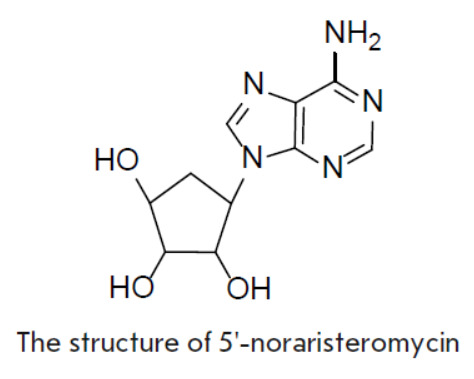



The structure of 5'-noraristeromycin

## EXPERIMENTAL

**Materials and Methods**

5′-Noraristeromycin as a racemic mixture of enantiomers was synthesized according to the procedure described in [13].

**Cell lines**

The Jurkat, K562, and THP-1 cells were cultured using RPMI 1640 medium (Gibco, USA) supplemented with 10% fetal bovine serum (FBS), 100 μg/mL penicillin, 100 μg/mL streptomycin, 1 mM sodium pyruvate, and 2 mM *L*-glutamine. The cell cultures were incubated at 37°C in the presence of 5% CO_2_.

**Cytotoxicity of 5′-noraristeromycin against Jurkat, K562, and THP-1 leukemia cells**

To assess the cytotoxic activity of 5**′**-noraristeromycin on the THP-1, Jurkat, and K562 leukemia cells, they were seeded into the wells of a 96-well plate (2,500, 2,000, and 2,500 cells per well, respectively). The cells were then treated with the drug within a broad range of concentrations (0.86–50 μM). DMSO at a concentration of 0.25% per well (corresponding to the percentage of DMSO when administering the drug at maximum concentration) was used as the control. The total volume of the well was 100 μL. The cells were incubated for 72 h. Cell survival was evaluated using a Resazurin Cytotoxicity Assay Kit (CEL-04-4-30 ML) (Abisense, Russia). Resazurin was added to PBS at a 1 : 100 ratio (volume, 100 μL) and incubated at +37°C in the presence of 5% CO_2_ for 4 h. Absorbance was then measured (absorbance at 570 nm; reference wavelength, 620 nm) using an Multiskan FC microplate photometer (ThermoScientific, USA). The average signal for the wells containing the medium only was subtracted from the value recorded for each well. Next, the data obtained regarding the concentration were normalized to the control and the *IC*50 value (half-maximal inhibitory concentration) was calculated using nonlinear regression. At least three replicates were made for each concentration. The *IC*50 value was calculated, and diagrams showing the dependence of living cells on drug concentration were plotted using the GraphPad Prism software v.8.4.3 (GraphPad Software, San Diego, USA).

**Toxicity of 5′-noraristeromycin for white outbred mice**

The toxicity of the compound was studied in compliance with the Guidelines for Conducting Preclinical Studies of Drugs [17].

Male and female white outbred mice weighing 25–30 g were used as biomodels. The animals were procured from the nursery at the Research Institute of Hygiene, Toxicology and Occupational Pathology, Federal Medical and Biological Agency of the Russian Federation.

The experimental and control groups consisted of four animals; the number of male and female animals in the batches was identical. The mice were randomly assigned to groups taking into account the absence of external signs of diseases and homogeneity of body weight (± 10%).

Doses within the range from 40 to 1 000 mg/kg were tested. The substance was administered intragastrically using a metal probe (0.05 mL per 10 g body weight). The control animals received an identical amount of the solvent (99% DMSO of compendial grade, JSC “Tatchempharmpreparaty”, Russia) via the same route.

After administration of the substance, the animals were followed up for 14 days; data on clinical manifestations of intoxication and deaths were documented. The lethal doses of the substance were calculated by probit analysis according to the procedure proposed by D.J. Finney using the Microsoft Excel 2013 software on day 14 post-administration [18].

The necropsy of the animals that had not survived was conducted shortly after their death; the macroscopic signs of the impact of the studied substances were documented. Internal organs (heart, lungs, liver, spleen, pancreas, kidneys, stomach, small and large intestine) were removed [19, 20] and subjected to a histological examination. The biomaterial was fixed in 10% neutral formalin for 4 days. Next, the samples were dehydrated using ascending alcohol series, bleached using chloroform in a Cytadel 2000 tissue processor (Shendon), and embedded into a Histomix paraffin medium (Biovitrum, Russia). Paraffin sections 4–5 μm thick were prepared using a Microm HM340E rotary microtome and mounted onto glass slides. For survey studies, the sections were subjected to hematoxylin and eosin staining using the conventional procedure [20].

The microsections were studied and photographed using an AxioScope A1 microscope (Carl Zeiss, Germany) equipped with an AxioCam MRc5 highresolution digital camera. The recorded images were analyzed using the ZENpro 2012 software (Carl Zeiss).

## RESULTS AND DISCUSSION

**Fig. 1:**
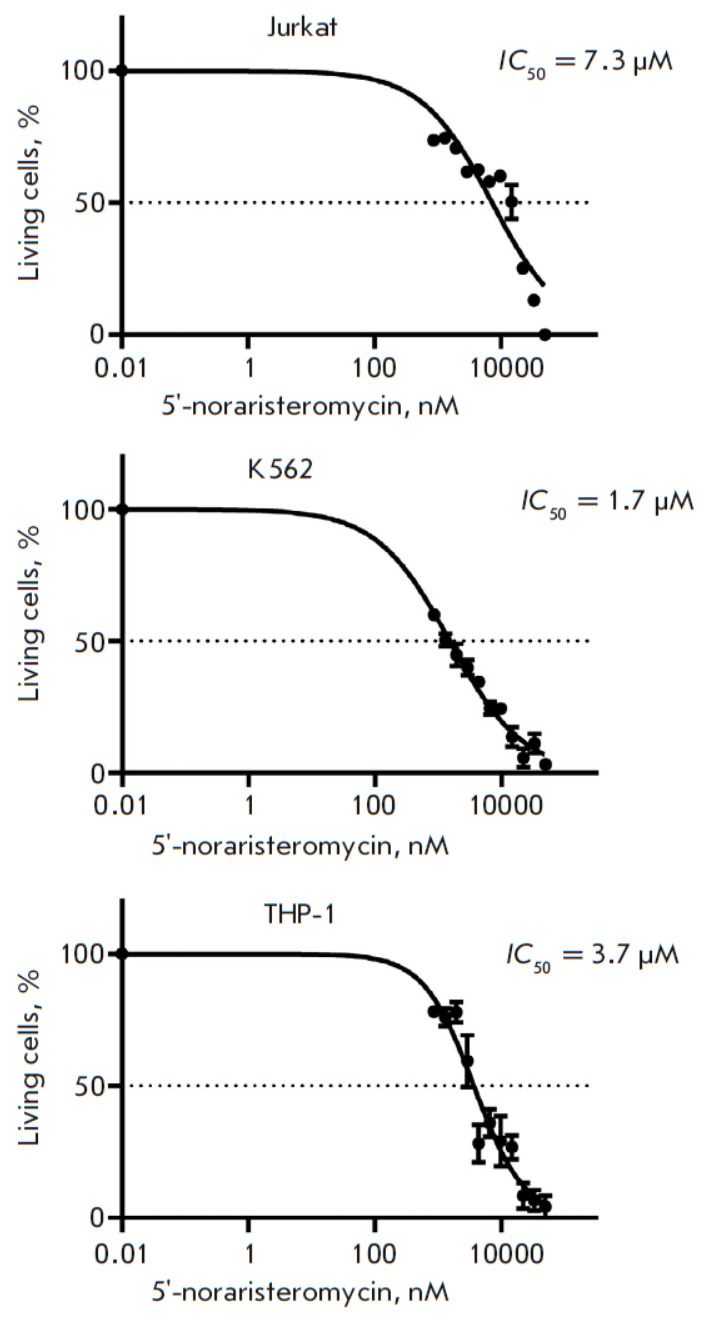
Survival of leukemia cells after treatment with 5′-noraristeromycin. Curves showing the percentage (%) of living Jurkat, K562, and THP-1 cells in wells treated with the drug at a concentration of 0.86–50 μM are shown. The *IC*50 values were calculated using nonlinear regression and are shown to the right of the graphs for each of the three cell lines

The cytotoxic activity of 5′-noraristeromycin was assessed in continuous Jurkat, THP-1, and K562 human leukemia cells. The drug was shown to exhibit a pronounced cytotoxic activity against all three cell lines. Significant cytotoxicity was observed for 5′-noraristeromycin concentrations < 1 μM. The half-maximal inhibitory concentrations (*IC*50) of the drug calculated using linear regression were as follows: ~7.3 μM for Jurkat cells, ~1.7 μM for K562 cells, and ~3.7 μM for THP-1 cells (*Fig. 1*). These values indicate that the drug is also potentially effective in models of laboratory animals, and it would be interesting to further study the mechanisms of its action against malignant leukemia cells.

*Table 1* summarizes the determined lethal doses of 5′-noraristeromycin administered intragastrically as a single dose.

**Table 1:** 5′-Noraristeromycin doses lethal to outbred mice after single-dose intragastric administration

Dose, mg/kg	Number of animals per group/number of nonsurviving animals	Calculated lethal doses, mg/kg		
		LD16	LD50 with confidence intervals	LD84
40	4/0	44.7	63.2 (52.7…75.8)	89.4
50	4/1			
80	4/3			
200	4/4			
1000	4/4			

Macroscopic assessment of the organs of nonsurviving animals showed that the main changes in them were those related to the gastrointestinal tract. At a dose ranging from 80 to 1 000 mg/kg, the drug caused hemorrhage into the gastric mucosa and the first part of the small intestine, resulting in thinning of their walls, sluggishness, and a yellowish-brown mucoid content in the small intestine. Sluggish small intestine with distended regions and yellowish-brown mucoid intestinal content were observed after the necropsy of the only nonsurviving animal that had received 50 mg/kg 5′-noraristeromycin.

**Fig. 2:**
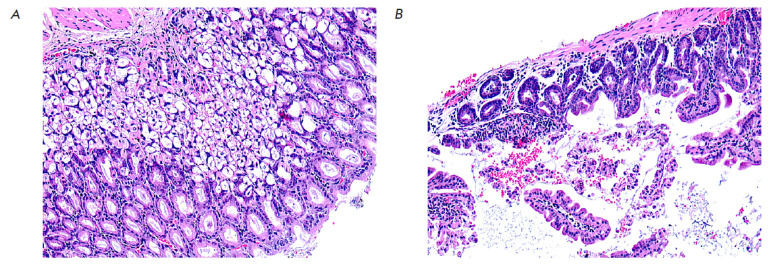
A fragment of the mucous membrane of the wall of the stomach (*A*) and small intestine (*B*) in an experimental mouse after administration of 80 mg/kg 5′-noraristeromycin. Hematoxylin–eosin staining. 200× magnification

Administration of 5′-noraristeromycin at a dose of 80 mg/kg, which is close to the median lethal dose, to the animals induced pronounced changes in the histoarchitectonics of the analyzed organs and tissues. Hence, microscopic examination of the gastric wall revealed an activation of chief cells and mucoid cells, accompanied by gland dilation and accumulation of mucoid contents in them (*Fig. 2A*). Death and desquamation of numerous mucosal epithelial cells into the intestinal lumen was found when examining a small intestine fragment. The remaining villi became more flattened; their apical surface contained no fringe of microvilli. Thinning of the submucosa and partial reduction of the muscle and serous membranes were also observed (*Fig. 2B*).

**Fig. 3:**
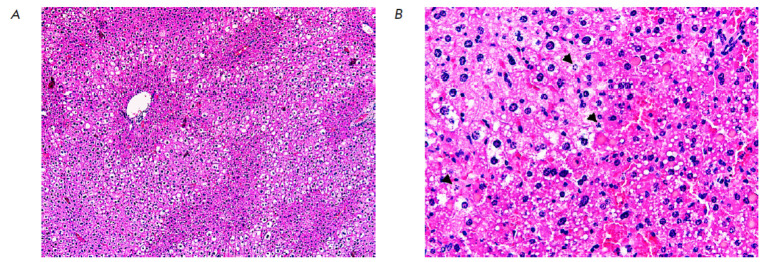
A fragment of liver tissue from an experimental mouse after administration of 80 mg/kg 5′-noraristeromycin. The hepatocytes with a fragmented nucleus are apoptotic cells (shown with an arrow); round cells with homogeneous cytoplasm are necrotic cells. Hematoxylin–eosin staining. Magnification ×100 (*A*); ×400 (*B*)

Total discomplexation of hepatic plates, severe periportal steatosis in combination with hepatocyte death in the centrilobular regions was observed in the liver tissue (*Fig. 3A*). Morphological examination revealed signs of apoptosis related to the death of some hepatocytes, while other cells had undergone necrotic changes (*Fig. 3B*). Either nuclear pyknosis or chromatin redistribution into the submembrane space was observed in relatively intact hepatocytes.

**Fig. 4:**
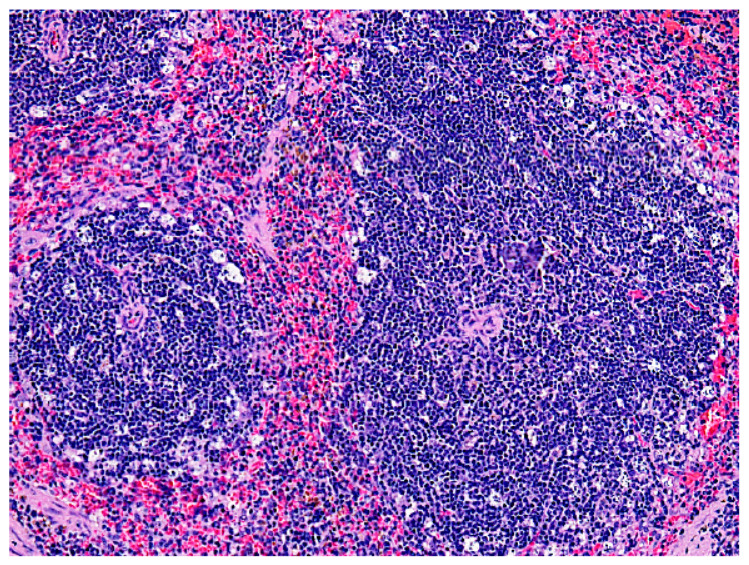
A fragment of the spleen of an experimental mouse after administration of 80 mg/kg 5′-noraristeromycin. Hematoxylin–eosin staining. 200× magnification

Death of multiple lymphoid cells at the periphery of white pulp follicles was detected in the spleens of the experimental animals (Fig. 4).

**Fig. 5:**
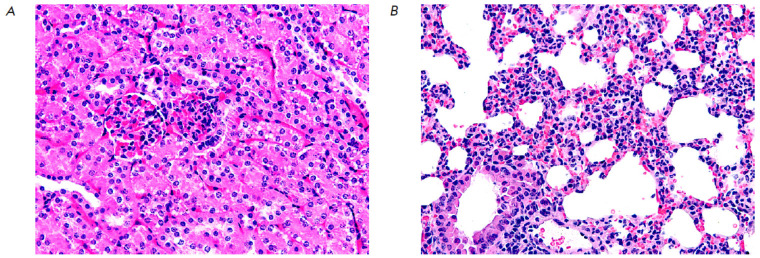
A fragment of kidney (*A*) and lung (*B*) tissue from an experimental mouse after administration of 80 mg/kg 5′-noraristeromycin. Hematoxylin–eosin staining. 400× magnification

Erythrocyte stasis and sludging in glomerular capillaries and the intertubular space were observed in the kidneys of an experimental mouse. The lumens of numerous convoluted tubules had narrowed, because of the hypertrophy of the epithelial cells lining them; the cytoplasm contained multiple vacuoles (*Fig. 5A*). Infiltration of the interstitial lung tissue by polymorphonuclear neutrophils was observed in the lung tissue of one experimental mouse. Alveolar septal thickening and edema, as well as erythrocyte diapedesis into interalveolar septa, were also detected (*Fig. 5B*).

Therefore, having studied the toxic properties of 5′-noraristeromycin, we determined that the median lethal dose of the compound administered orally to outbred mice was 63.2 mg/kg. The main intoxication symptoms within the first several minutes were neurological abnormalities (sudden agitation and hind limb dysfunction); the later symptoms (during the period between 2 h post-administration until death) involved severe hypodynamia.

Clinical manifestations and the results of the pathomorphological study demonstrated that administration of 5′-noraristeromycin at doses ≥50 mg/kg causes systemic damage to the internal organs and tissues of experimental animals. The negative effect was primarily noted in the organs of the gastrointestinal tract (stomach, small intestine, and liver) and the immune system (spleen).

Intragastric administration of 40 mg/kg 5′-noraristeromycin caused neither clinical signs of intoxication nor death of the experimental animals during the entire observation period.

## CONCLUSIONS

Comprehensive analysis of the study results revealed a high *in vitro *cytostatic activity of the newly synthe sized chemical compound, 5′-noraristeromycin, against Jurkat, K562, and THP-1 leukemia cells. Cytotoxic activity of the compound against leukemia cells was observed at concentrations as low as 1–10 μM. This compound had no toxic effect when administered intragastrically at a dose < 50 mg/kg. It is noteworthy that some drugs widely used in the therapy of malignant hematologic diseases have similar toxicometric parameters. An example is etoposide: according to different estimates, the range of half-maximal effective concentrations of this drug added to continuous leukemia cell lines is ~ 10–100 μM [21, 22]. Meanwhile, the nonlethal doses used in mouse model studies are ~ 50 mg/kg [23, 24].

In the experiments involving laboratory animals, a number of features of the general toxicity of the compound have been revealed in the range of lethal doses. 5′-Noraristeromycin will further require preclinical investigation and an assessment of specific antitumor activity in *in vivo *models to potentially develop a novel drug.

The data on the cytotoxic potential of the tested compound against leukemia cells obtained in preliminary studies, as well as the results of a study of general toxicity in laboratory animals, provide grounds to consider 5′-noraristeromycin as a promising antitumor agent. The mechanism of its cytotoxic activity against malignant cells and its suitability as a component in combination therapy along with the commonly used chemotherapeutics need more thorough study.

## Abbreviations

DMSO: dimethyl sulfoxide
IC50: half-maximal inhibitory concentration
LD16, LD50, and LD84: doses causing death in 16, 50, and 84% of experimental animals upon intragastric administration on day 14 of observation, mg/kg
PBS: phosphate-buffered saline
